# How Fast Do “*Owls*” and “*Larks*” Eat?

**DOI:** 10.3390/nu15061437

**Published:** 2023-03-16

**Authors:** Ludovica Verde, Annamaria Docimo, Giovanni Chirico, Silvia Savastano, Annamaria Colao, Luigi Barrea, Giovanna Muscogiuri

**Affiliations:** 1 Department of Public Health, University of Naples Federico II, 80138 Naples, Italy; 2 Centro Italiano per la Cura e il Benessere del Paziente con Obesità (C.I.B.O), Dipartimento di Medicina Clinica e Chirurgia, Unità di Endocrinologia, Diabetologia e Andrologia, Università degli Studi di Napoli Federico II, Via Sergio Pansini 5, 80131 Naples, Italy; 3 Dipartimento di Medicina Clinica e Chirurgia, Unità di Endocrinologia, Diabetologia e Andrologia, Università degli Studi di Napoli Federico II, Via Sergio Pansini 5, 80131 Naples, Italy; 4 Cattedra Unesco “Educazione alla Salute e allo Sviluppo Sostenibile”, University Federico II, 80138 Naples, Italy; 5 Dipartimento di Scienze Umanistiche, Università Telematica Pegaso, Via Porzio, Centro Direzionale, Isola F2, 80143 Naples, Italy

**Keywords:** eating speed, chronotype, obesity, fast eating, eating behaviors

## Abstract

Chronotype is a reflection of an individual’s preference for sleeping, eating and activity times over a 24 h period. Based on these circadian preferences, three chronotype categories have been identified: morning (MC) (*lark*), intermediate (IC) and evening (EC) (*owl*). Chronotype categories have been reported to influence dietary habits; subjects with EC are more prone to follow unhealthy diets. In order to better characterize the eating habits of subjects with obesity belonging to three different chronotype categories, we investigated eating speed during the three main meals in a population of subjects with overweight/obesity. For this purpose, we included 81 subjects with overweight/obesity (aged 46.38 ± 16.62 years; BMI 31.48 ± 7.30 kg/m^2^) in a cross-sectional, observational study. Anthropometric parameters and lifestyle habits were studied. Chronotype score was assessed using the Morningness–Eveningness questionnaire (based on their scores, subjects were categorized as MC, IC or EC). To investigate the duration of main meals, a dietary interview by a qualified nutritionist was conducted. Subjects with MC spend significantly more time on lunch than subjects with EC (*p* = 0.017) and significantly more time on dinner than subjects with IC (*p* = 0.041). Furthermore, the chronotype score correlated positively with the minutes spent at lunch (*p* = 0.001) and dinner (*p* = 0.055, trend toward statistical significance). EC had a fast eating speed and this, in addition to better characterizing the eating habits of this chronotype category, could also contribute to the risk of developing obesity-related cardiometabolic diseases.

## 1. Introduction

According to the latest reports of the World Health Organization (WHO), obesity is on an unstoppable increase worldwide, tripling in the last forty years [1]. The increased health risks brought on by excess body weight make it a major global health issue. It is known that obesity is a multifactorial disease in which lifestyle, socio-economic, genetic, endocrine, and several other factors intervene [2]. Despite the confirmed associations between obesity and its markers, there is an ongoing search for new factors to manage and slow the epidemic. Recent studies show that eating habits and obesity seem to be influenced also by chronotype [3,4,5], which reflects an individual’s preference for the timing of sleeping, eating, and activity in a 24 h period [6]. Morning, evening, and intermediate chronotypes are the three main categories of chronotypes. Subjects with morning chronotype (also called “*lark*”) prefer to rise early and tend to carry out activities earlier in the day. Contrarily, subjects with evening chronotype (also called “*owl*”) typically wake up later and prefer to schedule their peak activity during the late afternoon or evening. Between the morning and evening chronotypes, there are subjects with intermediate chronotype. Subjects with the intermediate chronotype tend to have a circadian rhythm that is somewhat more flexible with mixed characteristics. Therefore, they have greater flexibility in adapting to the demands of daily life, without any particular time slot being particularly problematic for them [6].

Nutrients, metabolic processes, and circadian rhythms are all interconnected. Because subjects with evening chronotype tend to engage in nighttime behaviors that are not in tune with the cycle of light and dark, they frequently experience circadian misalignment. Delay in meal timing and a habit of skipping breakfast has been often detected in subjects with evening chronotype [4]. In addition, engagement with excessive consumption during nighttime, lower protein and vegetables intake, as well as increased sucrose, sweets, caffeine, and alcohol intake characterize these subjects [4,7]. Probably as a consequence of this circadian misalignment and unhealthy lifestyle habits, subjects with evening chronotype have been reported to have more health problems, such as psychological disorders, gastrointestinal diseases, and higher mortality than the morning chronotype. Finally, evening chronotype has also been identified as a risk factor for the development of obesity-related cardiometabolic diseases [8,9]. In contrast, subjects with morning chronotype seem to have better, more protective dietary behavior, as indicated by regular consumption of breakfast and fresh and minimally processed foods, such as fruit, vegetables, fish, and dairy products [7].

As well as chronotype, eating behavior plays a crucial role in obesity-related cardiometabolic disease prevention and management due to its influence on energy balance and close interaction with other factors, such as nutrigenomics and psychology [10]. Eating behaviors are defined as the broad spectrum of behaviors related to eating lifestyle, including mealtimes, food preparation methods and eating speed. Of note, eating speed is one of the elements that accompanies the lives of modern consumers under time pressure. Therefore, consumers worldwide choose easy-to-prepare, often highly processed meals and spend long hours in front of screens both at work and in their leisure time. In this context, previous studies have shown that slow eating correlates with lower energy intake [11]. Actual evidence has also indicated the importance of slowing down consumption as a necessary factor in improving eating behavior [11]. Therefore, slow eating could be correlated with a lower incidence of obesity [10]. Many experimental studies have found that eating speed is correlated with obesity risk [10]. In a feasibility study in 21 participants randomly assigned to consume a 600 kcal meal at either a “normal” or “slow” rate (6 versus 24 min), Hawton et al. showed that slow eating could influence satiety, appetite and play a role in hormonal pathways [12]. Taking this evidence into account, an increasing number of studies have examined the interaction between eating speed and metabolic syndrome, diabetes, non-alcoholic fatty liver disease and cardiovascular disease [13,14,15]. All results showed a positive relationship between eating speed and these diseases and highlighted the need to analyze them in relation to obesity.

Since subjects with evening chronotype are more likely to present unhealthy eating habits, while subjects with morning chronotype are more likely to have healthy and protective habits, we aimed to better characterize nutritional habits of subjects with overweight/obesity belonging to the different chronotype categories, investigating the eating speed during the three main meals.

## 2. Materials and Methods

### 2.1. Design and Setting

This cross-sectional, observational research included participants from the Obesity, Programs of nutrition, Education, Research and Assessment of the best treatment (OPERA) project as well as patients at the Federico II University Hospital’s Endocrinology/Obesity Outpatient Clinic in Naples, Italy [16]. The research was authorized by Federico II University’s Ethical Committee (n. 5/14) and was carried out in accordance with the Declaration of Helsinki, which is the code of ethics for human experimentation adopted by the World Medical Association. All research participants received a thorough explanation of the study’s purpose, and their written informed consent was obtained. Recruitment consisted of an informational interview in which the details of the research were explained to the subjects, and they were encouraged to participate in the study.

### 2.2. Participants

Eligible participants for the study were adult subjects aged 18–75 years with normal liver, cardiopulmonary and kidney function as determined by interview. Additionally, we excluded patients who were taking medications for cardiovascular, renal, or pulmonary illnesses. Trained nutritionists assessed anthropometric parameters and asked standard questions including demographic information, personal medical history and lifestyle habits.

### 2.3. Anthropometric Assessment

The assessment period was from 8 to 12 a.m. Following an overnight fast, measurements were taken from each individual. The same qualified nutritionist conducted the anthropometric measurements. The subjects were measured wearing light clothing and without shoes. Height was measured with a stadiometer placed on a wall. Body weight was calculated with a calibrated scale. According to the National Center for Health Statistics [17], waist circumference was measured to the nearest 0.1 cm using a non-elastic tape measure at the natural indentation or at a point midway between the lower edge of the rib cage and the iliac crest if no natural indentation was evident. The hip circumference was measured while the subject was standing on one side and the measurement was taken from the widest point. For both measurements, subjects were standing with feet close together, arms along the sides and body weight evenly distributed. The subject had to be relaxed, and the measurements were taken at the end of a normal exhalation. Each measurement was repeated twice; if the measurements did not differ by more than 1 cm from each other, the average was calculated. If the difference between the two measurements exceeded 1 cm, the two measurements were repeated. The waist circumference was then divided by the hip circumference to determine the waist-to-hip ratio.

### 2.4. Lifestyle Habits

Participants who regularly exercised at least 30 min per day (YES/NO) were defined as physically active, as we have reported in detail in previous studies [5,8,15].

### 2.5. Eating Speed Assessment

As we have reported in detail in a previous study, eating speed was assessed for each main meal (breakfast, lunch and dinner) [15]. A face-to-face interview with a qualified nutritionist was performed to collect information about meal duration (minutes) and eating habits (habitual consumed foods and beverages) at the main meals (breakfast, lunch, and dinner). According to median value of meal duration, subjects were classified into two groups based on the following meals duration: fast eating group (FEG) (breakfast < 10 min, lunch < 20 min, and dinner < 20 min) or slow eating group (SEG) (breakfast  ≥ 10 min, lunch ≥ 20 min, and dinner  ≥ 20 min).

### 2.6. Chronotype Assessment

The Morningness–Eveningness Questionnaire (MEQ) was used to determine the chronotype of the subjects [18]. The MEQ consists of 19 multiple-choice questions about sleep patterns and daily functioning, including when participants feel most productive in physical or mental tasks, most tired, and most energized. Individuals were classified as morning (59–86), intermediate (42–58), or evening (16–41) chronotypes based on the sum of their individual items, which gave a total score ranging from 16 to 86 [18].

### 2.7. Statistical Analysis

Statistical analysis was performed according to standard methods using the Statistical Package for Social Sciences software 26.0 (SPSS/PC; SPSS, Chicago, IL, USA). The Kolmogorov–Smirnov test was used to assess the data distribution, and data that were not normally distributed were normalized using the logarithm. While categorical variables were expressed as frequency or percentage (N, %), continuous variables with normal distribution were given as mean ± standard deviation. One-way ANOVA and unpaired Student’s *t* test were used to analyze differences between groups (FEG and SEG and chronotype categories) for continuous variables. Chi-square test (χ^2^) was used to analyze differences between groups (FEG and SEG) for categorical factors. Using Pearson r correlation coefficients, we examined the relationships between chronotype score and eating speed during the major meals. The *p* values were considered significant at *p*  <  0.05.

## 3. Results

The main clinical characteristics of the study population are reported in Table 1. Eighty-one participants (28 men and 53 women) were included in the analyses. They were aged 46.38 ± 16.62 years and presented a mean BMI of 31.48 ± 7.30 kg/m^2^. Mean waist circumference of included subjects was 99.42 ± 20.64 cm while mean waist-to-hip ratio was 0.92 ± 0.13. Only a small fraction of the subjects was physically active (14, 17.3%).

### 3.1. Chronotype Categories in the Study Population and Degree of Obesity According to the Same Categories

**Figure 1:**
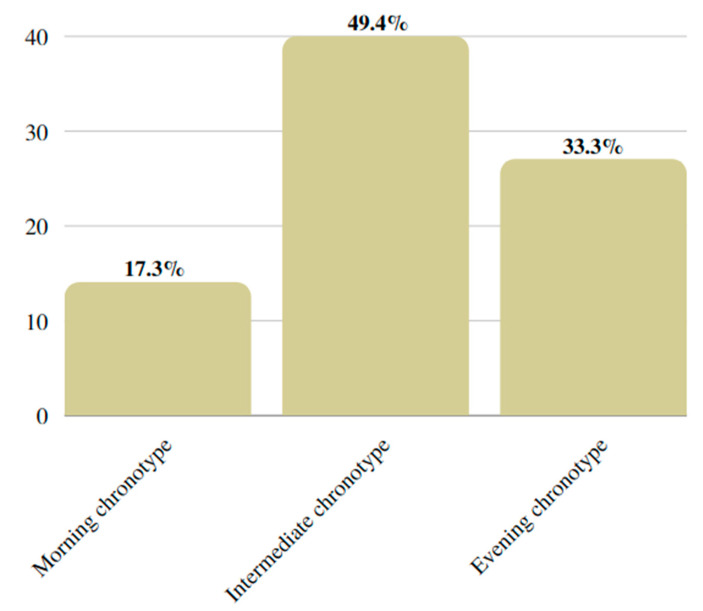
Study population divided into chronotype categories.

Chronotype categories in the study population were distributed as follows: 14 (17.3%) subjects had morning chronotype, 40 (49.4%) had intermediate chronotype, and 27 (33.3%) had evening chronotype (Figure 1).

**Figure 2:**
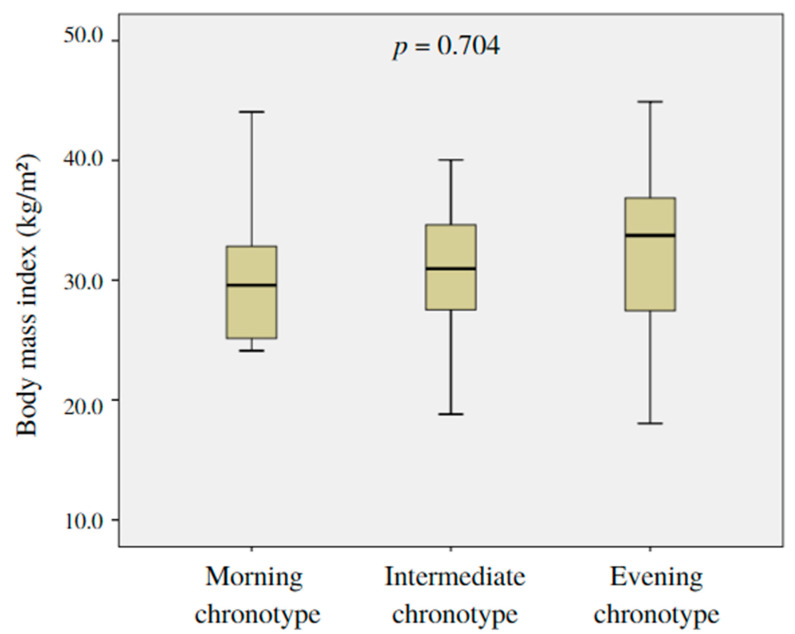
Mean body mass index according to chronotype categories.

The mean BMI of subjects divided into chronotype categories were as follows: 30.22 ± 5.53 kg/m^2^ for morning chronotype, 31.12 ± 6.01 kg/m^2^ for intermediate chronotype, and 32.00 ± 7.82 kg/m^2^ for evening chronotype. No differences were observed in BMI between the chronotype categories (*p* = 0.704) (Figure 2).

### 3.2. Clinical Characteristics According to Eating Speed at Main Meals

Table 2 shows the main characteristics of the entire study population according to the eating speed at breakfast, lunch and dinner. No significant differences were observed in terms of gender proportion, age, anthropometric parameters, and physical activity between FEG and SEG at breakfast, lunch and dinner. The only exceptions were a significantly higher waist circumference in the SEG group compared to the FEG group at lunch (0.96 ± 0.13 vs. 106.60 ± 11.27 cm; *p* = 0.041) and a significantly higher waist-to-hip ratio in the SEG group compared to the FEG group at both lunch (0.96 ± 0.13 vs. 0.90 ± 0.12; *p* = 0.041) and dinner (0.96 ± 0.13 vs. 0.89 ± 0.12; *p* = 0.039).

### 3.3. Chronotype Categories According to Eating Speed at Main Meals

Table 3 shows the differences in chronotype categories according to eating speed during the three main meals. No significant differences were observed in terms of chronotype categories between FEG and SEG at breakfast. At lunch, there was a significant difference in chronotype categories between FEG e SEG (*p* = 0.025). The same difference was observed at dinner describing a *p* value (0.050) close to but not quite statistically significant as supporting a trend toward statistical significance.

### 3.4. Differences in Eating Speed of Main Meals between Chronotype Categories

In particular, subjects with morning chronotype spend significantly more time on lunch than subjects with evening chronotype (18.93 ± 5.94 vs. 12.96 ± 6.39 min; *p* = 0.017) and significantly more time on dinner than subjects with intermediate chronotype (19.64 ± 5.71b vs. 15.25 ± 4.52; *p* = 0.041) (Table 4).

### 3.5. Correlation Studies

Simple and after-adjustment for BMI and age correlations between the chronotype score and the minutes spent eating breakfast, lunch and dinner are shown in Table 5. The chronotype score correlated positively with the minutes spent at lunch (*p* = 0.001) and dinner (*p* = 0.055, trend toward statistical significance). The positive correlation with minutes spent at lunch was maintained even after correction for confounding factors (*p* = 0.002)

## 4. Discussion

This cross-sectional observational study provides the first evidence to support the different eating speeds between the chronotype categories in subjects with overweight/obesity. In particular, the main finding of our study was that subjects with the morning chronotype spent significantly more time at lunch than subjects with the evening chronotype and significantly more time at dinner than subjects with the intermediate chronotype. In addition, the chronotype score was positively correlated with the minutes spent at lunch, i.e., as the time spent eating meals increases, the score indicating a morning chronotype increases (and vice versa).

There is a growing interest in the behavioral phenotypes linked to the development of obesity, with the purpose of better detailing the aspects on which we can intervene to improve the current treatment of this condition and its cardiometabolic complications. Eating speed has attracted attention as an indicator of appetite and satiety: a fast eating speed is thought to indicate greater motivation to eat, and more rapid deceleration over the course of the meal is thought to indicate a stronger response to internal satiety signals [19]. Experimental studies that manipulated eating speed in a controlled setting confirmed that fast eating speed is associated with greater food intake [20,21], and this higher food intake, certainly in conjunction with many other factors, could facilitate the development of obesity. In our population of subjects with overweight/obesity, this seems to be confirmed, as the FEG group is superior in absolute numbers for all three main meals, suggesting that a fast eating speed is a characteristic often present in individuals suffering from obesity.

Of interest, eating speed has a substantial heritable component, as reported in a study of twin pairs where the heritability of eating speed was high (0.62; 95% CI: 0.45, 0.74) [22]. In addition, research in animal models [23] and humans [24] has found that circadian rhythms are also partly genetically determined. The length of an individual’s circadian period can be significantly shorter or longer than the average 24 h period [25]. The extremes of this normal variation appear to be strongly driven by genetics, along with other features of circadian periodicity in humans; sensitivity to zeitgebers (environmental stimuli that synchronize the biological rhythms of organisms with their environment) has also been shown to be genetically variable [26]. Individual variations in phase, phase entrainment angle and period, together with environmental factors and age, contribute to the so-called “chronotype”, each person’s unique daily activity pattern. In the general population, these strong genetic determinants are presumably the sum of many small-effect genetic variations [24]. It has already been reported in the literature that subjects with evening chronotype have an overall unhealthier lifestyle. Subjects with an evening chronotype are more frequently smokers, sedentary, consume more processed foods and have a lower adherence to the Mediterranean diet [27]. These and other evening chronotype-related factors could contribute to the risk of developing obesity-related cardiometabolic diseases [9,28,29]. Our study adds a new element to this disadvantageous picture of the evening chronotype, that is, a fast eating speed. This, according to what has been previous reported, could be part of a partly genetically predetermined set-up that includes both chronotype and eating speed.

In particular, our results show that subjects with evening chronotype had a faster eating speed at lunch than subjects with morning chronotype. This is probably because lunch is, by social habits, the main meal in the study population (Italy), and since the evening chronotype is marked by poorer eating habits, it is likely to be characterized by a frugal meal, made with ready-to-eat or fast-food meals. We would also like to emphasize the fast eating speed at dinner of subjects with intermediate chronotype compared to subjects with morning chronotype. The intermediate chronotype is less described in the literature, and the limited evidence on it indicates that this chronotype category has mixed general characteristics. However, in our study, subjects with intermediate chronotype seemed to have an eating speed more similar to that of subjects with evening chronotype, and thus representing almost half of our population, it could be another category of subjects that should be given more attention during clinical evaluations.

Finally, we found a positive correlation between chronotype score and minutes spent eating lunch. This reinforced the fact that in obesity, subjects with a lower chronotype score (i.e., those who had a greater preference for nighttime versus daytime activities) tended to eat faster at lunch than those with a higher chronotype score. Although this emphasizes the importance of expanding assessments of the eating behaviors of subjects with obesity, the correlation does not provide cause-and-effect relationships, and further studies are needed to replicate the data found in the current study.

The limitations of our study should be noted. We had no data on meal size; thus, the fast eaters could be such because they consumed smaller meals than the slow eaters. However, we did not observe differences in BMI; thus, we did not expect major differences in the meals (quality and quantity) consumed between the two groups. Another limitation of the study is that it did not include a “chewing count” for each bite of food or a timekeeping of the meals. However, the introduction of the counting or timing could have influenced the participants’ chewing or eating speed, moving away from a real-life study. For this reason, the most reliable way to assess eating speed was retrospectively, i.e., asking the person to remember how long it took them to eat a meal they had already eaten, as previously reported [15]. This mode of assessment was more reliable, as there was no possibility of the subjects altering their eating behavior, knowing that they were being watched or timed.

A strength of our study is the novelty of the discovery, which anticipates future research in the field of the behavioral aspects that characterize obesity. Since it does not seem that obesity will stop growing, further efforts are certainly needed to intervene on new factors that favor the development of this pathology. Evening chronotype as well as eating speed are both well-known predisposing factors for the development of obesity-related cardiometabolic diseases. Finding new solutions to modify these behavioral aspects could play an important role in future anti-obesity complications strategies. Further study is required to develop effective interventions to manipulate eating speed over the long-term by using a variety of techniques, such as environmental changes, behavioral training beginning in infancy, and changes to food texture.

## 5. Conclusions

The findings of this study contribute to a better characterization of the eating habits of subjects with the evening chronotype, who were shown to eat faster than subjects with the morning chronotype. As these connections have an influence on the possibility of developing cardiometabolic diseases, the interaction between chronotype and eating speed in subjects with obesity is significant. It is crucial to understand which aspects of this eating behavior determine health risk and how this translates to people with circadian timing abnormalities, including evening chronotypes and shift workers, as epidemiological studies show that evening chronotypes are at increased risk of obesity and chronic diseases. This could ultimately pave the way for new behavioral interventions in their management.

## Figures and Tables

**Table 1:** Main characteristics of the study population.

Parameters	Subjects (*n* = 81)
Gender (M/F)	28/53
Age (years)	46.38 ± 16.62
BMI (kg/m^2^)	31.48 ± 7.30
Waist circumference (cm)	99.42 ± 20.64
Hip circumference (cm)	107.96 ± 12.19
Waist to Hip ratio	0.92 ± 0.13
Physical activity (yes)	14 (17.3%)

Data are expressed as mean ± standard deviation or number and (percentage). BMI, body mass index.

**Table 2:** Main characteristics of the study population according to eating speed.

Parameters	Fast Eating Group	Slow Eating Group	*p* Value
	**Breakfast**		
	<10 min (*n* = 42)	≥10 min (*n* = 39)	
Gender (M/F)	13/29	15/24	0.478
Age (years)	49.69 ± 16.85	42.82 ± 15.82	0.128
BMI (kg/m^2^)	32.33 ± 7.54	30.56 ± 7.00	0.334
Waist circumference (cm)	97.76 ± 19.57	101.21 ± 21.85	0.419
Hip circumference (cm)	107.98 ± 11.61	107.95 ± 12.93	0.951
Waist-to-hip ratio	0.90 ± 0.12	0.93 ± 0.13	0.214
Physical activity (yes)	4	10	0.055
	**Lunch**		
	<20 min (*n* = 53)	≥20 min (*n* = 28)	
Gender (M/F)	19/34	9/19	0.739
Age (years)	48.30 ± 16.98	42.75 ± 15.58	0.255
BMI (kg/m^2^)	31.23 ± 6.74	31.96 ± 8.36	0.774
Waist circumference (cm)	95.96 ± 18.41	105.96 ± 23.27	**0.041**
Hip circumference (cm)	106.60 ± 11.27	110.54 ± 13.60	0.198
Waist to hip ratio	0.90 ± 0.12	0.96 ± 0.13	**0.041**
Physical activity (yes)	7	7	0.182
	**Dinner**		
	<20 min (*n* = 52)	≥20 min (*n* = 29)	
Gender (M/F)	18/34	10/19	0.990
Age (years)	47.31 ± 17.02	44.72 ± 16.06	0.626
BMI (kg/m^2^)	31.61 ± 7.41	31.24 ± 7.21	0.874
Waist circumference (cm)	96.54 ± 19.25	104.58 ± 22.34	0.085
Hip circumference (cm)	107.31 ± 12.44	109.14 ± 11.85	0.503
Waist to hip ratio	0.89 ± 0.12	0.96 ± 0.13	**0.039**
Physical activity (yes)	7	7	0.223

Data are expressed as mean ± standard deviation or number and (percentage). Differences between groups were analyzed by unpaired Student’s *t* test (for continuous variables) or chi-square (χ^2^) test (for categorical variables). BMI, body mass index. A *p* value in bold type denotes a significant difference (*p* < 0.05).

**Table 3:** Differences in chronotype categories according to eating speed during the three main meals.

	**Fast Eating Group**	**Slow Eating Group**	***χ2***	***p* Value**
	**Breakfast**			
	<10 min	≥10 min		
Morning chronotype	5	9	2.8505	0.240
Intermediate chronotype	20	20		
Evening chronotype	17	10		
	**Lunch**			
	<20 min	≥20 min		
Morning chronotype	5	9	7.3614	**0.025**
Intermediate chronotype	27	13		
Evening chronotype	21	6		
	**Dinner**			
	<20 min	≥20 min		
Morning chronotype	5	9	5.9752	0.050
Intermediate chronotype	28	12		
Evening chronotype	19	8		

Data are expressed as number of subjects. The chi-square (χ^2^) test was used to determine the significance of differences in chronotype categories between the two groups of eating speed during the three main meals. A *p* value in bold type denotes a significant difference (*p* < 0.05).

**Table 4:** Differences in eating speed of main meals between chronotype categories.

Minutes Spent at	Morning Chronotype (*n* = 14)	Intermediate Chronotype (*n* = 40)	Evening Chronotype (*n* = 27)	*p* Value
Breakfast	9.64 ± 4.99	8.00 ± 4.36	7.59 ± 4.24	0.361
Lunch	18.93 ± 5.94 ^a^	15.00 ± 6.10	12.96 ± 6.39	**0.017**
Dinner	19.64 ± 5.71 ^b^	15.25 ± 4.52	15.56 ± 6.98	**0.041**

^a^ vs. evening chronotype and ^b^ vs. intermediate chronotype. Data are expressed as mean ± standard deviation. One-way ANOVA was used to determine the statistical significance of differences in minutes spent during the three main meals between chronotype categories. A *p* value in bold type denotes a significant difference (*p* < 0.05).

**Table 5:** Correlations between chronotype score and eating speed at the main meals.

Minutes Spent at	Chronotype Score			
	Simple Correlation		after Adjusted for BMI and Age	
	*r*	*p* Value	*r*	*p* Value
Breakfast	0.185	0.099	0.098	0.392
Lunch	0.370	**0.001**	0.347	**0.002**
Dinner	0.214	0.055	0.187	0.099

A *p* value in bold type denotes a significant difference (*p* < 0.05). BMI, body mass index.

## Data Availability

The data presented in this study are available on request from the corresponding author.
